# Ultra Short Heart Rate Variability as a Prognostic Marker in Pulmonary Embolism: A Retrospective Cohort Study

**DOI:** 10.3390/jcm15041488

**Published:** 2026-02-13

**Authors:** Shay Perek, Majd Lahham, Tarek Arraf, Naama Sitry, Khalil Hamati, Yori Gidron, Ayelet Raz-Pasteur

**Affiliations:** 1 Department of Emergency Medicine, Rambam Health Care Campus, 8 Ha’Aliyah Street, Haifa 3109601, Israel; s_perek@rambam.health.gov.il; 2 The Ruth and Bruce Rappaport Faculty of Medicine, Technion, Israel Institute of Technology, 1 Efron Street, Haifa 3525433, Israel; 3 Department of Internal Medicine A, Rambam Health Care Campus, 8 Ha’Aliyah Street, Haifa 3109601, Israel; majd.lahham91@gmail.com (M.L.); t_arraf@rambam.health.gov.il (T.A.); 4 Maccabi Health Services, North District, Haifa 3308148, Israel; 5 The Gastroenterology Institute, Rambam Health Care Campus, 8 Ha’Aliyah Street, Haifa 3109601, Israel; 6 Clalit Health Services, Department of Family Medicine, North District, Nazareth 1710601, Israel; naamasitry@gmail.com; 7 Azrieli Faculty of Medicine, Bar-Ilan University, Safed 1311502, Israel; 8 Meuhedet Health Services, North District, Haifa 3304508, Israel; khalilh.hamati@gmail.com; 9 Faculty of Welfare and Health Sciences, University of Haifa, Haifa 3103301, Israel; ygidron@univ.haifa.ac.il

**Keywords:** pulmonary embolism, ultra short heart rate variability, risk stratification, electrocardiogram, emergency department acute care, personalized medicine

## Abstract

**Background/Objectives**: Pulmonary embolism (PE) remains a significant cause of cardiovascular mortality, with risk stratification being critical for optimizing treatment decisions. Heart rate variability (HRV), a measure of autonomic nervous system function, had been explored as a prognostic index in various cardiovascular conditions, yet has received limited investigation regarding PE prognosis. Our objective was to evaluate the prognostic value of ultra-short HRV indices, obtained at the emergency department (ED), in patients presenting with PE. **Methods**: A retrospective cohort study, conducted at Rambam Health Care Campus, Haifa, Israel. All eligible patients diagnosed with acute PE at the ED, between the years 2010 and 2012 were included. Further, a subgroup analysis was performed to differentiate between oncological (*n* = 118) and non-oncological (*n* = 115) patient populations. Ten-seconds electrocardiogram was used to compute ultra-short HRV indices, specifically SDNN (standard deviation of normal-to-normal RR intervals) and RMSSD (root mean square of successive differences). Multivariate logistic regression models were created to assess HRV’s independent predictive value for 30-day and 90-day mortality. In addition, a survival analysis was carried out utilizing Cox regression and Kaplan-Meier curves. **Results**: 233 patients (42% male; age 65 ± 17) were included in the analysis. Ultra-short HRV indices did not significantly correlate with short-term mortality. However, in non-oncological patients (*n* = 115), multivariate analysis demonstrated that higher SDNN (as a continuous variable), was independently associated with increased 90-day mortality (AOR 1.018, 95% CI 1.000–1.037; *p* = 0.044). In contrast, HRV showed no predictive value for mortality in oncological patients. In both the entire cohort and the non-oncological sub-group, Kaplan-Meier plots established statistically significant differences, with lower HRV indices correlating with worse survival. This finding is paradoxical. The issue of context-dependent HRV (i.e., based on ECG obtained during rapid shallow breathing, which reduces HRV on the one hand, but is possibly adaptive during an acute PE, to increase oxygen supply and prevent shock in the short run, on the other hand), may explain these findings. **Conclusions**: Ultra-short HRV shows some promise in short term risk stratification of non-oncological PE patients. As for oncological patients, HRV was not found to have short term prognostic relevance.

## 1. Introduction

Venous thromboembolism (VTE), clinically presenting as deep vein thrombosis (DVT) or pulmonary embolism (PE), is the third leading cause of vascular death after myocardial infarction (MI) and cerebral vascular accident (CVA) and is the most common preventable cause of death in hospitalized patients. While the age-standardized mortality rate from MI and CVA declined in high-income countries over the past 2 decades, reports on mortality from VTE during the past twenty years vary with certain countries, with specific geographical and economic conditions, showing reduction in PE-related mortality, while in others, the rates remained stable [1,2,3]. The age-stratified crude PE-related death rate varies widely depending on patients’ age, with a recent significant increase in the younger age groups (i.e., 25–39 and 40–54 years old) [4]. This may be associated with the diagnostic challenge encompassed in PE, as demonstrated by the high rates of out-of-hospital fatal PE and in-hospital fatal PE diagnosed only after post-mortem [5]. Emergency department (ED) utilization of extensively validated clinical severity scores [e.g., Pulmonary Embolism Severity Index (PESI)] [6] or the combination of laboratory and radiological findings (e.g., troponin and right ventricular dysfunction observed on echocardiograms) [7], have been used for early risk stratification and the personalization of therapeutic decisions. The development and incorporation of additional prognostic markers could significantly enhance the correct ED classification of patients with PE, affecting patient care and disposition.

Electrocardiogram (ECG) is the staple of cardiovascular assessment in the ED. PE patients may exhibit ECG abnormalities (e.g.,; tachycardia, S1Q3T3 pattern, complete right bundle branch block, inverted T waves in leads V1-V4, ST elevation in lead aVR, and atrial fibrillation), which are associated with an increased risk of circulatory shock and death [8]. Heart rate variability (HRV), a measure of cardiac autonomic nervous system activity, mostly reflects vagal parasympathetic activity to the heart, and can be measured from ECG output. HRV has been extensively studied in various fields, including in cardiac patients, notably those surviving acute MI and congestive heart failure. Reduced HRV in these patient groups is associated with increased mortality risk [9]. Vagal nerve activity inhibits inflammation [10]. Inflammation has been shown to be related to hyper-coagulable states [11] and PE as well as right ventricular dysfunction [12]. In addition, posttraumatic stress disorder (PTSD) was found to increase DVT risk, with heightened stress-associated neural activity and lower HRV, mediating the effect of PTSD on DVT risk [13].

This suggests that HRV could also play an important role in patients presenting with acute PE, aiding in risk stratification. Reduced HRV time-domain indices derived from 24-h holter monitoring of patients with acute PE, were found to be associated with echocardiographic and biochemical signs of RV overload, as well as 30-day major adverse cardiovascular events [14,15]. Ultra-short HRV analysis, focusing on recordings under five minutes has shown strong correlations with longer recordings, especially for time-domain indices [16,17]. Ultra-short HRV obtained in the ED setting, has shown promise, as a means of risk stratification in other cardiovascular conditions [18,19]. Therefore, the goal of the current study was to evaluate the use of ultra-short HRV, derived from a standard ECG segment, and examine its prognostic significance in patients arriving at the ED with PE.

## 2. Materials and Methods

### 2.1. Study Design and Population

This retrospective cohort study was conducted at Rambam Health Care Campus (RHCC; Haifa, Israel) and included all eligible patients diagnosed with acute PE, at RHCC ED, between January 2010 and December 2012. The study was approved by the Institutional Review Board (Helsinki approval number 0603-16-RMB). Informed consent was waived due to the retrospective nature of the analysis. Patients aged ≥18 years with a confirmed PE diagnosis via CT pulmonary angiography or incidental PE detection on other CT scans were included. Exclusion criteria included PE diagnosed solely via lung scintigraphy, absence of ECG or HRV data, and ECGs with arrhythmias (e.g., atrial fibrillation) or poor resolution, preventing HRV analysis. Sub-group analysis (i.e., oncological vs. non-oncological patient groups), was based on patient oncological status at the time of ED visit.

### 2.2. Data Collection and Variables

Demographic, clinical, and laboratory data were collected from electronic medical records, utilizing MDClone software Version 6 (Beer-Sheva, Israel). Vital signs, laboratory markers (i.e., D-dimer, troponin, BNP), and relevant medications were recorded. Patients’ past medical history, with an emphasis on oncological status, was also documented. Missing data was not extrapolated and was left empty (i.e., “not available”).

### 2.3. ECG Analysis and Computation of Ultra-Short HRV Indices

Patients underwent a 10-s resting ECG (LAN Green model, Norav Medical, Yokne’am, Israel), while lying motionless in a supine position for at least 30 s. ECGs were obtained within 30 min from ED arrival, as part of our ED patient admission protocol. Patients in respiratory distress received oxygen prior to ECG analysis. The ECG electrodes were placed in anatomical positions according to standard procedure. An ECG viewing program was used to visualize the resting ECG files (Resting ECG version 5.62, Norav Medical). PR interval duration and QRS interval duration were automatically measured, and a corrected QT interval was calculated based on the Bazzett equation. In addition, HRV parameters were computed, utilizing a custom version of the HRV analysis software able to import and analyze 10-s-long recordings (HRV version 5.62, Norav Medical). Furthermore, ECGs were manually checked and recordings with disturbances (e.g., excessive noise, sudden baseline instability or low resolution), were excluded. ECGs which contained excessive premature ventricular or supraventricular activity (e.g., atrial fibrillation, atrial flutter, atrial tachycardia) as well as advanced atrio-ventricular conduction abnormalities (e.g., complete heart block or other high-degree conduction abnormalities) were also excluded. Linear time-domain HRV variables (e.g., standard deviation of RR intervals [SDNN] and root mean square of successive differences [RMSSD]) were assessed in our study, due to their high agreement with longer-term recordings. HRV indices were automatically calculated based on 10-s ECG strip. All ECGs were manually assessed in “HRV mode” of the Norav software, to make sure the correct R waves were identified and marked, thus allowing correct calculation of SDNN and RMSSD. The HRV indices were assessed both as continuous and categorical variables (e.g., greater than the median length).

### 2.4. Outcomes and Statistical Analysis

The primary outcomes were 30 and 90-day all-cause mortality. Secondary outcomes included overall survival analysis. Analysis was carried out on the entire cohort, and on two sub-groups (oncological and non-oncological patients). The study database was analyzed with R software (version 4.0.3, The R Foundation for Statistical Computing, Vienna, Austria). Continuous variables are presented as means and standard deviation (SD), unless otherwise specified. Categorical variables are presented as absolute numbers and percentages. Correlations between variables and Boolean outcomes were examined with logistic regressions (LR) and presented as odds ratio (OR) with 95% confidence intervals (95%CI) and *p*-values. Variables found to have statistical significance (*p*-value < 0.05) or trend (*p*-value < 0.1) in univariate analyses, were introduced into a multivariate LR model, in a backward stepwise fashion. Multivariate model accuracy is presented with receiver operating characteristic (ROC) curves, including the area under the curve (AUC). Survival analysis was carried out with Cox regression and presented with Kaplan-Meier curves as well.

## 3. Results

### 3.1. Patients’ Baseline Characteristics

**Figure 1:**
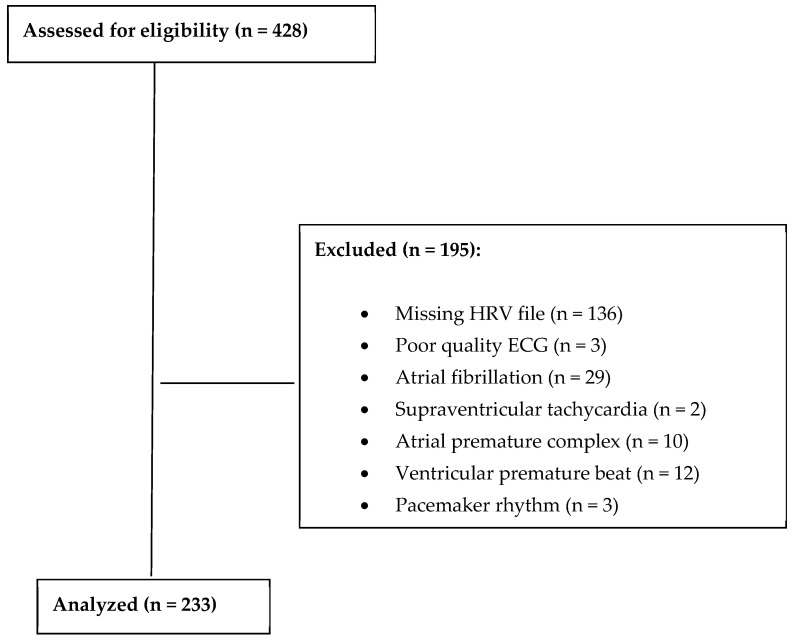
Patient enrollment flow diagram.

428 patients were assessed for eligibility. 195 of these patients were excluded. The final study cohort included 233 patients who had arrived at Rambam health care campus from January 2010 till December 2012 and were diagnosed with acute PE, as noted in Figure 1. Markedly, 56 (13%) of the patients were excluded due to arrhythmia. Patients’ characteristics are detailed in Table 1. The majority were female, over half were suffering from malignancy at the time of their ED visit, and 6 patients had a history of thrombophilia. The Charlson Comorbidity Index, available for 203 patients, had a mean score of 7 ± 3, reflecting a high burden of comorbidities. Notably, 31% (73 patients) had an oxygen saturation level of ≤92%, an indicator of potential respiratory distress.

### 3.2. HRV Indices Compared to Published Norms

HRV parameters in our study cohort were relatively low compared with published 10-s HRV values corrected for age and gender [20]. SDNN (median 17.4 (±25.9) ms, IQR 6.5 ms–25.5 ms) was found to be lower than median values in 76.0% of patients, with 41.3% of patients having SDNN values lower than the 2nd percentile of their age and gender-corrected range.

As for RMSSD (mean 23.0 (±34.9) ms, IQR 10.2 ms–35.8 ms), 74.6% of patients and 33.3% of patients had values lower than age and gender-corrected median and 2nd percentile, respectively.

### 3.3. HRV and Mortality (Entire Cohort n = 233)

**Figure 2:**
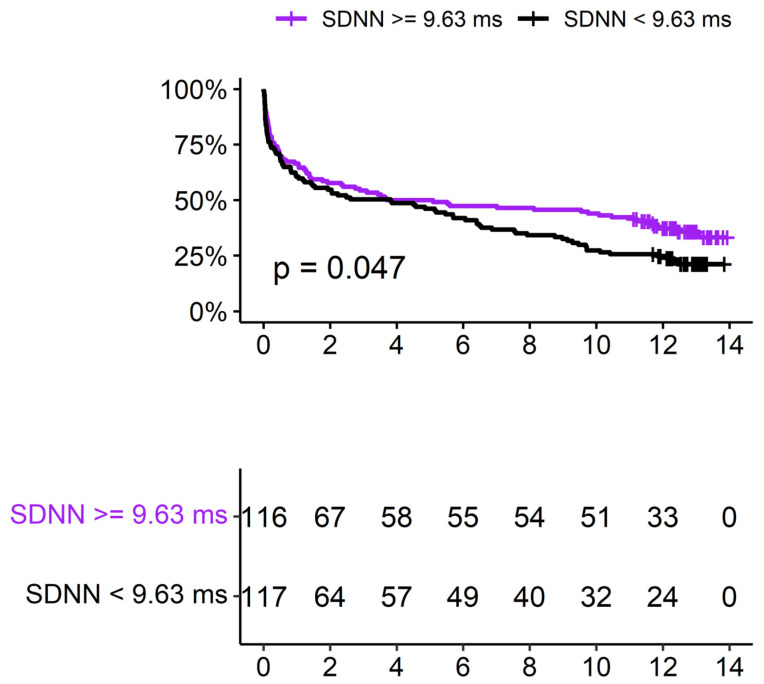
Kaplan-Meier curve and SDNN (entire cohort, *n* = 233).

Thirty-three (14%) patients died within 30 days of diagnosis. HRV was not found to be correlated with all-cause mortality (SDNN-OR: 1.003 (CI 0.991–1.016); *p*—0.575; RMSSD-OR: 1.004 (CI 0.996–1.013), *p*—0.281). In addition, 58 (25%) patients died within 90 days of diagnosis. Similarly, HRV was not found to be correlated with all-cause mortality (SDNN-OR: 1.001 (CI 0.990–1.012), *p*—0.827; RMSSD-OR: 1.002 (CI 0.994–1.010), *p*—0.556). Survival analysis (follow up interval—5.7 (±5.4) years; 165 (71%) patients died during the follow up period), revealed mixed results. While Cox regression did not demonstrate statistically significant correlations (SDNN-HR: 0.999 (CI 0.993–1.006), *p*—0.926; RMSSD-HR: 1.001 (CI 0.996–1.005), *p*—0.602), assessment of Kaplan-Meier curves exhibited curve separation after 6 years with lower HRV (less than the median of this study group; 9.63 ms), correlated with increased mortality (*p*—0.047), as presented in Figure 2.

### 3.4. Sub-Group Analysis

#### 3.4.1. Oncological Patients

One-hundred and eighteen of the study patients (51%) were suffering from an oncological disease when diagnosed with PE. This group was heterogenic-21 (18%) lung cancer, 16 (14%) breast cancer, 14 (12%) hematological malignancy, 11 (9%) colorectal cancer, 9 (8%) undifferentiated cancer, 7 (6%) stomach cancer, 7 (6%) skin cancer, 7 (6%) sarcoma, 6 (5%) gynecological malignancy, 5 (4%) pancreatic cancer, 5 (4%) prostate cancer, 3 (2%) liver cancer, 3 (2%) brain cancer, 2 (2%) thyroid cancer, 1 (1%) kidney cancer and 1 (1%) bladder cancer patients. 72 (61%) of the patients arrived at the ED with known cancer, diagnosed on average 212 (±1120) days prior to PE diagnosis. The remaining 46 patients were diagnosed during PE index admission. HRV indices were not found to be correlated with all-cause mortality both in short term intervals (30-days: SDNN-OR: 0.987 (CI 0.956–1.019), *p*—0.432; RMSSD-OR: 0.994 (CI 0.974–1.016), *p*—0.632 and 90-days: SDNN-OR: 0.976 (CI 0.948–1.006), *p*—0.128; RMSSD-OR: 0.989 (CI 0.971–1.008), *p*—0.273) and when assessing survival analysis during a follow up period of 3.4 (±4.8) years (SDNN-HR: 0.995 (CI 0.984–1.006), *p*—0.357; RMSSD-HR: 0.999 (CI 0.991–1.007), *p*—0.815).

#### 3.4.2. Non-Oncological Patients

One-hundred and fifteen of the study patients (49%) did not have underlying cancer when diagnosed with PE. 7 (6%) and 11 (10%) patients died within 30 and 90 days, respectively. When analyzing 90-day all-cause mortality, HRV indices were found to be correlated with mortality both in univariate and multivariate analysis, as detailed in Table 2. SDNN and RMSSD as univariate predictors, demonstrated ROC curve area under the curve (AUC) of 0.6327 and 0.6224, respectively. Multivariate model included 4 parameters: patient gender with men at increased risk, as well as higher Charlson and lower Norton scores were also correlated with increased mortality. Finally, SDNN was also part of the multivariate model. With constant gender, Charlson and Norton scores, an increase in SDNN of 1 ms was related with an increase of 1.8% in the risk of 90-day mortality. Multivariate model ROC curve demonstrated a high AUC-0.9091.

**Figure 3:**
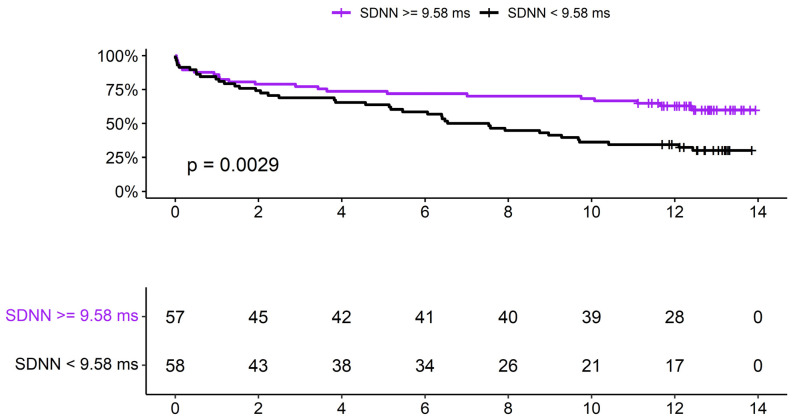
Kaplan-Meier curve and SDNN (non-oncological cohort, *n* = 115).

While Cox regression did not demonstrate statistically significant correlations between HRV indices and survival (SDNN–HR: 1.003 (0.995–1.011), *p*—0.373; RMSSD–HR: 1.003 (0.997–1.009), *p*—0.279), Kaplan-Meier curves (follow up of 8.1 (±5.1) years) disjoined after 4 years with a *p*-value of 0.0029, as shown in Figure 3. SDNN lower than 9.58 ms (cohort median) was associated with lower survival over time.

#### 3.4.3. HRV Comparison of Oncological vs. Non-Oncological Groups

HRV time domain indices, did not differ between the two sub-groups. SDNN for the oncological group was 9.58 (IQR 6.65–17.16) ms, compared with 9.60 (IQR 6.66–17.14) for the non-oncological group (*p*-value 0.685). As for RMSSD, for the oncological group was 13.59 (IQR 8.14–23.15) compared with 13.45 (IQR 8.02–23.12) for the non-oncological group (*p*-value 0.704).

## 4. Discussion

This study explored the prognostic role of ultra-short HRV indices in ED patients diagnosed with PE. Low ultra-short HRV was found to be a significant predictor of long-term mortality in the overall cohort and among the non-oncologic patient subgroup. Conversely, among non-oncologic patients, higher SDNN and RMSSD values were associated with increased 30-day and 90-day mortality.

The low SDNN, a surrogate of HRV, reflects the appropriate sympathetic dominance as an adaptive response in acute PE. This sympathetic activation is needed to maintain cardiac output, support systemic blood pressure and allows for compensation of increased right ventricle systolic pressure, thus representing a focused, efficient autonomic response, rather than autonomic failure.

Several studies demonstrated improved prognosis with higher HRV, both in cardiovascular illness and cancer [21]. In contrary, patterns, in which higher HRV values were associated with worse clinical outcomes, have been demonstrated in other conditions such as pneumonia [22] and infective endocarditis [23]. Furthermore, in patients with sepsis, elevated HRV components, were paradoxically associated with greater illness severity and worse clinical outcomes [24,25]. The common feature across these mentioned studies, may be that patients presenting with high vagal activity upon hospital arrival already have an underlying inflammatory condition, to which the cholinergic anti-inflammatory vagal pathway is responding. As in infectious processes, an inflammatory response is present. Increasing evidence supports a pathophysiological association between inflammation and thrombosis. In the context of infection, sepsis, pneumonia, endocarditis and in pulmonary embolism, inflammation may lead to a compensatory increase in vagal activity—reflected by elevated HRV—in an attempt to activate the cholinergic anti-inflammatory reflex. Such an increase in HRV, rather than being protective, may therefore represent a maladaptive or insufficient compensatory response and could predict a poorer prognosis in these inflammatory conditions [26].

A possible hypothesis explaining our findings relates to the acute setting of patients’ diagnosis at the ED. The ECG from which HRV was derived was obtain in the ED, when patients were suffering from acute PE. The context in which the ECG was performed, from which HRV was derived, included rapid shallow breathing, to compensate for lack of oxygen. Such rapid shallow breathing reduces HRV on one hand [27], but is possibly adaptive during an acute PE, leading to increased oxygen supply and shock prevention in the short run, on the other hand. An inability to shift from vagal-parasympathetic slow paced breathing to sympathetic and rapid breathing in such a context, may reflect a non-adaptive response, which could predict then a poor outcome. Though in another domain unrelated to health, the precise effect of context on HRV can be seen in studies on HRV as a predictor of performance in the military. When HRV was derived hours prior to an extreme military exercise began (“hell week”), low HRV (reflecting a sympathetic shift) predicted better performance [28] In contrast, if resting HRV was derived long before severe military stress, high HRV predicted better outcomes, such as completion of the air-force course [29]. Thus, we suggest that low HRV during the acute phase on a PE may reflect adaptation to the PE and, if short-term, may then predict a better clinical outcome.

Another possibility is that inflammation may be a contributing factor for thrombus formation in part of our cohort. Inflammation has been shown to reduce HRV [30], thus altering or even reversing the potential prognostic role of HRV, especially in non-oncological patients with PE.

In contrast to non-oncological patients, ultra-short HRV did not demonstrate predictive value for mortality in cancer patients, a finding which diverges from what one might expect, given that cancer patients typically exhibit lower baseline HRV. Malignancy is associated with chronic autonomic dysfunction, likely driven by systemic inflammation, cachexia, and metabolic derangements. Previous studies have demonstrated that elevated inflammatory markers, such as interleukin-6 (IL-6) and C-reactive protein (CRP), inversely correlate with HRV, suggesting that systemic inflammation may suppress vagal tone and reduce HRV independently of PE [31]. As a result, autonomic alterations attributable to cancer may obscure or minimize additional HRV changes induced by acute PE. Moreover, cancer-related mortality may overshadow HRV’s prognostic significance in this population, as malignancy itself is a major determinant of mortality, with multiple competing causes of death including disease progression, treatment-related toxicity, secondary infections, and metabolic complications. Finally, cancer therapies such as chemotherapy, radiotherapy, and analgesic treatments may further alter autonomic regulation, potentially confounding the relationship between HRV and PE outcomes in this subset of patients.

Our results differ from previously reported studies assessing HRV and PE prognosis. Right ventricle systolic pressure and SDNN were found to have negative correlations [14]. However, in our report, we did not measure RVSP, but focused only on mortality. While there is an association between RVSP and mortality, it is not the only factor which may lead to patient death [32]. As for the association between HRV and early mortality risk [15], the study focused on the comparison to a non-PE control group. In our cohort, we also demonstrated relatively low ultra-short HRV indices, when compared to published norms. Thus there is agreement with Mao et al.

One of the major strengths of this study is its large cohort of acute PE patients, including a significant number of oncological and non-oncological patients, allowing for subgroup analysis. Additionally, the use of ultra-short HRV analysis from 10-s ECGs ensures clinical applicability, as such recordings are widely available in primary care and emergency settings. However, there are several limitations to consider. The retrospective design of this study poses inherent constraints (e.g., large number of missing variables, inability to compare to existing risk stratification tools due to lack of specific variables), and prospective validation is needed. Furthermore, since only ultra-short HRV was captured and calculated, we could focus solely on time-domain indices, as frequency domain parameters are invalid in these very short time intervals. Moreover, our sample size and relatively low number of outcome events, did not allow for a validation cohort. In addition, our long survival analysis time period was selected as we aimed to also assess HRV and survival over long time intervals. Nonetheless, during the past 15 years, the field of PE management has changed with the introduction of direct oral anticoagulants (DOACs) as a viable alternative to heparin and warfarin. DOACs’ efficacy has been shown to be as good as low molecular weight heparin and warfarin, with lower adverse effects (e.g., bleeding) [33]. This may potentially have a positive effect on patient morbidity and mortality. Therefore, our mortality rates may be higher than expected rates today, thus diminishing our study’s generalizability potential. Furthermore, overfitting might have occurred with our non-oncological group multivariate model for 90-day all-cause mortality, due to the low number of positive events (i.e., death within 90 days). Notably, RMSSD and SDNN as univariate parameters, demonstrated some prediction capabilities. Finally, our decision to exclude ECGs with arrhythmia, due to the inability to calculate ultra-short HRV indices, may have introduced a selection bias. 56 patients (of the original 428 patients assessed for eligibility; 13%) were excluded due to an arrhythmia. Notably, the lack of arrhythmia may indicate our study cohort represents a more challenging subset of PE patients, with regard to clinical decision making. Thus, the addition of HRV to other risk stratification tools, may provide additional support for optimal acute management.

## 5. Conclusions

Ultra-short HRV shows some promise in short term risk stratification of non-oncological PE patients. As for oncological patients, HRV was not found to have short term prognostic relevance. External validation and comparison to existing PE specific risk stratification tools is in order. Future studies should focus on refining HRV-based risk models and explore their integration into PE management algorithms and therapeutic strategies.

## Figures and Tables

**Table 1:** Patients’ characteristics (*n* = 233).

	Average/Number	Standard Deviation/Percent
Age (years)	65	±17
Gender—male	97	42%
Ethnicity—Jewish; Arab	132; 33	57%; 14%
Height (cm; *n* = 93)	161	±31
Weight (kg; *n* = 89)	79	±17
Thrombophilia	6	3%
Malignancy	118	51%
Charlson score (*n* = 203)	7	±3
Norton score (*n* = 230)	16	±4
Emergency department—vital signs		
Systolic Blood Pressure (mmHg)	138	±27
Diastolic Blood Pressure (mmHg)	78	±14
Pulse (beats-per-minute)	94	±18
Shock index	0.71	±0.20
Saturation ≤ 92%	73	31%
Temperature (PO; °C)	36.7	±0.4
Emergency department—blood work		
White blood cells (×10^3^/µL)	12	±20
Hemoglobin (g/dL)	12	±2
Platelets (×10^3^/µL; *n* = 228)	238	±110
Creatinine (mg/dL)	1.1	±0.6
Glucose (mg/dL)	138	±67
pH (*n* = 128); HCO_3_ (mmol\L; *n* = 128)	7.40; 23.5	0.04; 3.5
PT (seconds; *n* = 158); PTT (seconds; *n* = 157)	12.0; 28.2	3.6; 6.4
D-Dimer (mg/L; *n* = 41)	2.48	±1.79
Troponin (ng/mL; *n* = 114)	0.18	±0.66
Emergency department—ECG & ultra-short HRV		
PR interval (ms)	160	±33
QRS interval (ms)	88	±19
QTC interval (ms)	434	±33
SDNN (ms)	17.4	±25.9
RMSSD (ms)	23.0	±34.9

SDNN—Standard deviation of RR intervals; RMSSD—Root mean square of successive differences.

**Table 2:** Non-oncological patients-90-day all-cause mortality logistic regression (*n* = 115).

	Univariate Analysis		Multivariate Analysis	
	OR (95% CI)	*p*-Value	AOR (95% CI)	*p*-Value
Age (years)	1.042 (1.000–1.085)	0.048 *		
Male gender	3.936 (0.986–15.700)	0.052	7.806 (1.326–45.927)	0.023 *
Charlson score	1.642 (1.190–2.265)	0.002 *	1.666 (1.124–2.471)	0.011 *
Norton score	0.789 (0.678–0.917)	0.002 *	0.811 (0.667–0.987)	0.037 *
Saturation < 90%	3.500 (0.970–12.627)	0.055		
SDNN (ms)	1.015 (1.002–1.029)	0.017 *	1.018 (1.000–1.037)	0.044 *
RMSSD (ms)	1.011 (1.001–1.020)	0.021 *		

SDNN—Standard deviation of RR intervals; RMSSD—Root mean square of successive differences. * *p*-value < 0.05.

## Data Availability

The raw data supporting the conclusions of this article will be made available by the authors on request.

## Abbreviations

The following abbreviations are used in this manuscript:

PE	Pulmonary embolism
HRV	Heart rate variability
ED	Emergency department
SDNN	Standard deviation of normal-to-normal RR intervals
RMSSD	Root mean square of successive differences
AOR	Adjusted odds ratio
VTE	Venous thromboembolism
DVT	Deep vein thrombosis
MI	Myocardial infarction
CVA	Cerebrovascular accident
PESI	Pulmonary embolism severity index
ECG	Electrocardiogram
PTSD	Post-traumatic stress disorder
RHCC	Rambam health care campus
SD	Standard deviation
LR	Logistic regression
OR	Odds ratio
ROC	Receiver operating characteristic
AUC	Area under curve
HR	Hazard ratio
IL-6	Interleukin 6
CRP	C-reactive protein
